# Trial Protocol: Communicating DNA-based risk assessments for Crohn's disease: a randomised controlled trial assessing impact upon stopping smoking

**DOI:** 10.1186/1471-2458-11-44

**Published:** 2011-01-19

**Authors:** Sophia CL Whitwell, Christopher G Mathew, Cathryn M Lewis, Alastair Forbes, Sally Watts, Jeremy Sanderson, Gareth J Hollands, A Toby Prevost, David Armstrong, Ann Louise Kinmonth, Stephen Sutton, Theresa M Marteau

**Affiliations:** 1 Health Psychology Section, Department of Psychology, King's College London, 5th Floor Bermondsey Wing, Guy's Campus, London SE1 9RT, UK; 2 Department of Medical and Molecular Genetics, King's College London School of Medicine, 8th Floor Tower Wing, Guy's Hospital, Great Maze Pond, London SE1 9RT, UK; 3 Department of Gastroenterology and Clinical Nutrition, University College Hospital, 235 Euston Road, London NW1 2BU, UK; 4 Clinical Genetics, 7th Floor New Guy's House, Guy's Hospital, London SE1 9RT, UK; 5 St Thomas Hospital, Lambeth Palace Road, London SE1 7EH, UK; 6 King's College London, Department of Primary Care and Public Health Sciences, 5th Floor Capital House, 42 Weston Street, London SE1 3QD, UK; 7 University of Cambridge Department of Public Health and Primary Care, Forvie Site, Robinson Way, Cambridge, CB2 0SR, UK

## Abstract

**Background:**

Estimates of the risk of developing Crohn's disease (CD) can be made using DNA testing for mutations in the NOD2 (CARD15) gene, family history, and smoking status. Smoking doubles the risk of CD, a risk that is reduced by stopping. CD therefore serves as a timely and novel paradigm within which to assess the utility of predictive genetic testing to motivate behaviour change to reduce the risk of disease. The aim of the study is to describe the impact upon stopping smoking of communicating a risk of developing CD that incorporates DNA analysis. We will test the following main hypothesis:

Smokers who are first degree relatives (FDRs) of CD probands are more likely to make smoking cessation attempts following communication of risk estimates of developing CD that incorporate DNA analysis, compared with an equivalent communication that does not incorporate DNA analysis.

**Methods/design:**

A parallel groups randomised controlled trial in which smokers who are FDRs of probands with CD are randomly allocated in families to undergo one of two types of assessment of risk for developing CD based on either:

i. DNA analysis, family history of CD and smoking status, or

ii. Family history of CD and smoking status

The primary outcome is stopping smoking for 24 hours or longer in the six months following provision of risk information. The secondary outcomes are seven-day smoking abstinence at one week and six month follow-ups. Randomisation of 470 smoking FDRs of CD probands, with 400 followed up (85%), provides 80% power to detect a difference in the primary outcome of 14% between randomised arms, at the 5% significance level.

**Discussion:**

This trial provides one of the strongest tests to date of the impact of communicating DNA-based risk assessment on risk-reducing behaviour change. Specific issues regarding the choice of trial design are discussed.

**Trial Registration:**

ISRCTN: ISRCTN21633644

## Background

There are high expectations regarding the potential for estimates of disease risk incorporating DNA analysis to motivate behaviour change more strongly than other types of risk information [1,2]. Such expectations are consistent with theories of attitude change which predict that the greater the personal salience of information, such as information regarding one's own DNA, the greater the impact [3]. We present here a protocol for a randomised controlled trial assessing the behavioural impact of using DNA analysis to estimate disease risk. This DNA analysis will be used to quantify the susceptibility to Crohn's disease (CD) of smokers who are first degree relatives (FDRs) of probands with CD, a susceptibility that is modifiable by smoking cessation. The behavioural effect of communicating the results of the analysis will be measured by the proportion of smokers who report stopping for 24 hours or longer in the six months following the provision of the risk assessment.

CD is a relatively common, complex genetic condition with a population prevalence of around 1 per 1000 per lifetime [4] and first degree relatives (FDRs) have an approximately twenty-fold increased risk of developing the condition [5]. Results from recent epidemiological and genetic studies now make it possible to offer relatives of probands increasingly precise information about their chances of developing the disease, using DNA analysis of the NOD2 genotype [5,6].

Smoking is an additional risk factor and is associated with a two-fold increase in the risk of developing CD [7]. Smoking also leads to a more aggressive course in those with the disease [8,9]. Importantly, this pattern is reversed by smoking cessation [10].

As can be seen from Table 1 the relative risks for NOD2 mutations in CD are much lower than the risks for highly penetrant mutations in single gene disorders. However, they are higher than most other mutations associated with complex disorders. In those who are NOD2 mutation-negative the lifetime absolute risk of developing CD is very low (approximately 2% in first degree relatives; see Lewis et al [5]). Since commercial testing is currently being offered for a range of common, complex disorders with the expectation of motivating behaviour to reduce risk (e.g. 23 and Me https://www.23andme.com/; Navigenics http://www.navigenics.com/ predictive testing for CD serves as a timely and novel paradigm in which to assess the impact of communicating the results of predictive genetic testing in motivating risk-reducing behaviour change, in this case smoking cessation. A series of genome-wide association scans in CD have recently identified multiple new susceptibility genes and loci (reviewed by Mathew [11]). In most cases, however, the associations have been detected with single nucleotide polymorphisms (SNPs) that tag common haplotypes at these loci. Since most of the causal genes or causal sequence variants have not yet been clearly defined, the precise degree of additional genetic risk that they confer is unknown.

**Table 1:** Estimated frequencies of NOD2 genotypes in probands and FDRs, and estimated FDRs' probability of developing CD.

	NOD2 mutation negative	NOD2 mutation heterozygous	NOD2 mutation homozygous
**Frequency of NOD2 genotype in CD probands**	65%	27%	8%

**Frequency of NOD2 genotype among FDRs**	75%	22%	3%

**Frequency of NOD2 genotype in controls**	87%	12%	1%

**Risk to FDRs of developing CD conferred by genotype**	2%	4%	15%

We are unaware of any previous attempt to provide relatives of probands with CD with information that might encourage them to engage in behaviours to reduce their risks of developing the condition. Of interest is whether providing precise and personalised DNA-based information about the likelihood of developing Crohn's disease and how such a likelihood might be reduced leads to smoking cessation. Two studies have evaluated the impact of DNA-based risk information of developing lung cancer on smokers' motivation to stop smoking. Both used a general population sample [12-14]. While the first of these provided evidence of increased motivation to stop smoking [12,13], the other did not, with over 50% of smokers in the study failing to understand or recall their DNA test results [14]. Neither study found that DNA-based risk information increased smoking cessation.

There is some evidence to suggest that when DNA analyses reveal no risk-enhancing mutations there may be higher levels of false reassurance and hence lower rates of risk-reducing behaviour than similar levels of risk estimated without DNA analyses [15,16]. Such an effect may follow from presenting the results of the analysis of a risk factor that is personally salient and perceived as categorical *i.e*. the mutation is either present or it is not, although the risk it confers is most often probabilistic. We therefore predict that those whose risk analysis includes DNA but for whom no risk-enhancing mutations are found will be less likely to make a quit attempt than those whose risk analysis did not include DNA analysis. Additionally, we predict a dose-response effect of the receipt of DNA-based risk information: individuals who are revealed to have one or more risk-enhancing mutations will be more motivated to change their behaviour than those who receive a DNA-based risk revealing no mutations, or those in the comparison arm who did not undergo DNA analysis.

### Objective and hypotheses

The trial objective is to estimate the impact upon stopping smoking of communicating a risk of CD that incorporates DNA analysis. The specific hypotheses to be examined are as follows:

#### Hypothesis I (Main hypothesis)

Smokers who are FDRs of CD probands are more likely to make smoking cessation attempts following communication of risk estimates of developing CD that incorporate DNA analysis, compared with an equivalent communication that does not incorporate DNA analysis (between arm comparison).

#### Hypothesis II

Among smokers whose CD risk assessment includes feedback of DNA analysis, smoking cessation attempts are more likely when the analysis reveals one or more risk increasing mutations, when compared with (a) those undergoing a similar DNA analysis that does not reveal any risk increasing mutations (any versus no mutation; within DNA arm comparison), (b) risk assessment that does not incorporate DNA analysis (any mutation versus non-DNA arm).

#### Hypothesis III

Among smokers whose CD risk assessment includes feedback of DNA analysis, smoking cessation attempts are less likely when the analysis does not reveal any risk increasing mutations, when compared with smokers receiving a risk assessment that does not incorporate DNA analysis (no mutation versus non-DNA arm).

## Methods/Design

### Trial design (*see *Figure 1)

**Figure 1:**
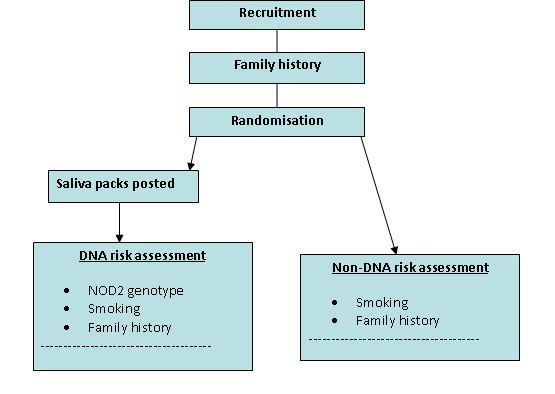
**Trial design**.

A parallel group cluster randomised controlled trial in which smokers who are first degree relatives of probands with Crohn's disease will receive the results of one of two types of risk assessment for developing Crohn's disease, based on:

i. DNA testing for NOD2 genotype, family history of CD and smoking status, or,

ii. family history of CD and smoking status

### Participants

Participants will comprise 470 FDRs of probands with CD, aged 18 years and over, who do not have a diagnosis of CD or ulcerative colitis and who smoke five or more cigarettes daily. Participants must be able to give informed consent and to complete, either alone or with assistance, the study questionnaires.

Exclusion criteria:

1. Cigar, pipe and oral tobacco users who do not also smoke five or more cigarettes daily.

2. Those currently taking medication for smoking cessation or medication with a known influence on smoking cessation that they cannot stop (e.g. nortriptyline for depression).

3. Those who are non-English speakers.

### Interventions

The components of the interventions described below are shown in Table 2.

**Table 2:** Intervention components present in DNA and non-DNA risk assessment arms

Intervention component	DNA arm	Non-DNA arm
Mouthwash posted to participant to collect saliva sample for NOD2 Genotyping	√	X

Booklet containing results of risk assessment posted approximately 6 weeks after initial contact	√	√

Personal risk presented using a numerical risk with a denominator of 1000, accompanied by a visual display	√	√

Feedback of risk assessment based on family history of CD and smoking Status	√	√

Feedback of risk assessment based on NOD2 genotype	√	X

Explanation of NOD2 gene in booklet	√	X

Explanation of how stopping smoking reduces the risk of CD in booklet	√	√

Smoking cessation advice over the telephone	√	√

#### Communication of risk assessment for Crohn's disease

All participants will receive a booklet outlining the results of their risk assessment of developing CD. This comprises presentation of three lifetime risk figures: population risk (1:1000); personal risk, presented numerically with a denominator of 1000 and accompanied by a visual display [17]; and personal risk following smoking cessation. The booklet includes explanations of how the risk figure was estimated and of how stopping smoking reduces the risk of CD. The nature of the risk assessment differs by arm and risk estimates are calculated as follows:

#### Comparison (non-DNA) arm

Personal risks of developing CD are based on residual familial risk i.e. whether the proband is a parent, sibling or child (estimating the effect of unidentified genes, after accounting for the contribution of NOD2); and smoking status (two-fold increased risk for smokers), as described in previous research [5].

#### Intervention (DNA) arm

Personal risks of developing CD are again based on residual familial risk and smoking status, plus NOD2 genotype (conferring a gene dosage effect on risk) derived from a mouthwash sample returned by the participant, again calculated according to prior research [5]. In effect, the risk communicated to the DNA arm is calibrated into high, medium and low, whilst the control arm receive an "averaged" genetic risk, based on their family history. DNA will be extracted and analysed for known NOD2 (also known as CARD15) susceptibility mutations (*see Procedure for more details*).

#### Brief smoking cessation advice

Receipt of the results booklet is followed by a telephone call (see "Telephone call 2" in Procedure) from a research counsellor trained as an NHS Stop Smoking Service provider who goes over the information in the booklet to ensure comprehension and to deliver a brief smoking cessation intervention. This is aimed at increasing motivation to stop smoking and in those motivated to stop, to encourage use of the NHS Stop Smoking Services.

### Procedure

#### Participant recruitment

First degree relatives of people affected by Crohn's disease will be identified via three routes:

i. Probands receiving care through hospital services. Proband addresses will be obtained by gaining accesses to CD proband databases at participating hospitals, following the approval of the local hospital Trust Research and Development department. Probands may be given study invitation packs or telephoned by members of the clinical team.

ii. Probands who are members of the National Association for Colitis and Crohn's Disease (NACC). All members who have CD will be posted an invitation pack.

iii. Advertising in the newsletters of NACC and the charity Ostomy Lifestyle.

Probands will be posted a study information booklet and consent form. They will be asked to inform any FDRs of theirs who smoke and who are unaffected by CD or ulcerative colitis about the study. They will be asked to indicate on the consent form their preferred method for the study team to contact their relatives, either requesting the study information from the research team in order to give this to their relatives themselves, or providing their relatives' contact details, for the research team to make the contact. This procedure was developed as part of a feasibility study for this trial [18].

Information booklets and consent forms will be distributed to identified FDRs. Eligible FDRs who have read and understood the study information booklet will sign and return the consent form to the research team at KCL. The research counsellor will then telephone the participant and obtain further verbal consent before entering them into the trial.

#### Telephone call 1

Information will be collected about the participant's family history of CD to contribute to the risk assessment, as well as demographic and smoking characteristics. The participant's date of birth will be noted for randomisation at a later date.

Participants will be randomised after Telephone call 1. Those randomised to the DNA arm will be asked to provide a saliva sample for a DNA test. Non-DNA arm participants will not undergo a DNA test and hence will not be asked to provide a saliva sample. The trial co-ordinator will then send the results booklet to the participant (see *Communication of risk assessment for Crohn's disease*).

#### Telephone call 2

Following receipt of the results booklet, the research counsellor will telephone the participant to administer the brief smoking cessation intervention as previously described.

#### Telephone call 3

A member of the research team who did not deliver the intervention will assess self-reported seven-day smoking cessation approximately one week after telephone call 2 has taken place.

#### Telephone call 4

This will take place six months after the second telephone call to assess the primary endpoint, seven-day smoking cessation, and levels of worry caused by the risk assessment. This telephone call will be made by a member of the research team who did not deliver the intervention and who is blind to allocation. Saliva samples will be collected via post in those who report seven day smoking abstinence, to allow cotinine validation.

#### DNA testing

DNA will be extracted and analysed for the three known NOD2 susceptibility mutations: R702W, G908R and 1007fs by allele-specific PCR amplification [19]. Assays are to be validated by the inclusion of individuals with known NOD2 genotypes derived by DNA sequencing in each genotyping experiment. A greater than 95% success rate is expected for the extraction and amplification of DNA. In the event that DNA is not collected or the PCR procedure fails, the participant will be asked to provide a further sample. While the prevalence of NOD2 mutations is lower or zero in populations not of European ancestry, the penetrance of the gene is not known to vary by ethnicity.

### Outcomes

#### Primary outcome

The primary outcome, assessed six months after the intervention, is making one or more quit attempts of 24 hours or longer in the six months following assessment of CD risk. This end point is a reliable predictor of eventual cessation [20].

#### Secondary cessation outcomes

##### Seven-day smoking cessation at one week

Self-reported smoking cessation will be assessed at one week following receipt of the CD risk assessment.

##### Seven-day smoking cessation at six months

At six months abstinence from smoking in the preceding seven days will be assessed using the Russell standard procedures [21], counting participants lost to follow up as being smokers, and self-reported smoking status will be verified biochemically. Validated abstinence requires smoking no more than 5 cigarettes in the prior seven-day period and a cotinine level of <15 ng/ml.

##### Additional outcomes

Questionnaires administered within telephone calls 1,3 and 4 will assess key variables derived from three psychological theories, specifically Leventhal's self-regulation model of health and illness [22], Protection Motivation Theory [23], and the Theory of Planned Behaviour [24]. We will also assess self-reported smoking-related behaviours (such as use of NRT) and participants' recall and comprehension of their risk assessment.

### Sample size

#### (a) Hypothesis I

The initial sample size estimate comprised 540 smokers who were FDRs of probands with Crohn's disease. 270 would be cluster randomised by family to DNA testing and 270 to the non-DNA arm. Allowing for 20% dropout we expected to follow-up 215 per arm. With this sample size, and allowing for clustering, there was 80% power to detect an odds ratio (OR) of 1.75 for likelihood of engaging in a quit attempt between randomised arms using a two-sided test at the 5% level of significance. In a general population sample without presentation of risk information 29% reported a quit attempt by six-month recall [25]. On this basis we have estimated that the proportion reporting having made one or more quit attempts of 24 hours or more in the preceding six months would be 35% in the non-DNA arm, enabling detection of 49% or higher in the DNA testing intervention arm (OR = 1.75). In order to allow for clustering effects by family the sample size was increased by a design effect of 8%. This was based on a mean cluster size of 1.13 from the pilot study [18] and an allowance that the intracluster correlation (ICC) could be as high as 0.6 based on observing a range of ICCs between 0.3 and 0.87 for behavioural outcomes clustered at the household level [26].

To check the assumptions underlying the sample size calculation, an interim analysis including the first 266 (50%) participants was conducted. This revealed a higher completion rate (87%), confirmed the cluster size (mean cluster size 1.14) and identified the ICC to be zero. The trial steering committee supported a revised sample size calculation based on a follow-up rate of 85% and without the need to account for clustering in the analysis. This meant that without loss of power for primary or secondary outcomes we could follow up 200 per arm, requiring recruitment of only 470 randomised participants.

#### (b) Hypotheses II and III

Amongst the 200 followed-up in the DNA analysis arm it is expected that 44 will be heterozygous for NOD2, six homozygous, and 150 mutation negative. The following sets of secondary comparisons will be made to test Hypotheses II and III:

II(a) Within DNA arm: 0 mutations vs. 1 or 2 mutations

II(b) Between non-DNA arm and a subgroup of DNA arm (1 or 2 mutations)

III Between non-DNA arm and a subgroup of DNA arm (0 mutations)

The odds ratios that can be detected with 80% power with the above group sizes at the 5% level of significance are as follows:

II(a): An average odds ratio of 2.5 within DNA arm both in the absence of any difference between the randomised groups (29% in 0 mutations group versus 52% in 1 or 2 mutations group; group average 35%; detectable OR = 2.55) and in the presence of a difference between the randomised groups (43% in 0 mutations group versus 65% in 1 or 2 mutations group; group average 49%; detectable OR = 2.45).

II(b): There is 80% power to detect an odds ratio of 2.5 between the non-DNA arm and those with 1 or 2 mutations in the DNA arm (35% in the non-DNA arm versus 57% in the 1 or 2 mutations group of the DNA arm; detectable OR = 2.45).

III: There is 80% power to detect an odds ratio of 0.5 between the non-DNA arm and those with 0 mutations in the DNA arm (35% in the non-DNA arm versus 21% in the 1 or 2 mutations group of the DNA arm; detectable OR = 0.50).

### Randomisation

#### (a) Procedure

The research counsellor (KCL) will enroll each participant into the trial and the trial coordination team (KCL) will send each participant's data for randomisation to the statistical team (Cambridge), and receive back the allocated group.

#### (b) Method

Each participant will be cluster randomised by family to one of the two arms, with allocation on a 1:1 basis. The allocation method will be blocked randomisation with the randomisation sequence prepared by the trial statistician.

#### (c) Concealment

The randomisation sequence will be concealed from the trial co-ordination team and research counsellor and the statistical team will only be given study identification data necessary for randomisation. Participant date of birth will be required at study closure to confirm agreement between the generated sequence and that used in the trial. After assignment of a participant, neither the research counsellor nor the participant will be blind to the participant's study arm.

### Research governance

COREC approval has been given (REC: Hertfordshire 1: 06/Q0201/19, 26^th ^June, 2006).

R&D approval has been obtained from the hospitals (42 NHS Trusts across the UK) through which the probands will be contacted, and from the South London and Maudsley Trust, the employer of the research counsellors.

### Fidelity checks

All telephone calls will be tape-recorded. A sub-sample of these recordings will be randomly selected and transcribed to assess fidelity to the clinical protocol. The tapes will be stored in a locked cabinet and a sample analyzed on a regular basis.

### Adverse events monitoring

All comments made by participants and health care professionals will be logged in the Trial Log File. They will be given an individual number and also logged on a computer database. Any follow-up or further action needed will also be logged. No personal information will be included unless absolutely necessary. The file will be stored in a locked office.

Any incident considered serious is to be entered in the Adverse Incident Log. This is a hardbound book that will also be stored in a locked office. A judgement about the incident's severity will be made between the Principal Investigator and the research team. Any incident included here will have a sheet in the Trial Log File and will also be logged on a computer database. Any follow-up or further action will be included in the book, alongside details regarding how the incident was resolved. No personal information will be included unless absolutely necessary.

An example of how adverse events monitoring could take place is as follows:

If a participant becomes anxious after learning of their risk of developing CD, the PI and research team will be informed and the incident logged. An appropriate procedure will then be initiated. Depending upon the nature of the anxiety, the participant will be encouraged to visit their GP or will be offered an appointment with of the trial's gastroenterology consultants. The outcome of this contact will be monitored and logged and the participant's GP informed, if consent is given for this.

Any incident judged to be severe by the Principal Investigator and the research team will be reported to the Data Monitoring and Ethics Committee within 24 hours.

### Statistical analysis

The analysis of the primary outcome, the proportion of participants reporting having made one or more quit attempts of 24 hours or more in the preceding six months, and the secondary cessation outcomes will be compared between arms by estimating a difference in proportions between arms together with a 95% confidence interval, and the p-value from the associated chi-squared test. The odds ratio and 95% confidence interval will also be reported. Analyses will be on an intention to treat basis with the common assumption for secondary cessation outcomes that all participants are classified as smokers except for those biochemically verified as non-smokers. However, for the primary outcome, those missing this outcome will primarily be treated as missing from the analysis. Missing status will be compared between groups, and the primary difference in proportions will be re-assessed assuming that missing data is imputed as no quit attempt. All tests will be two-sided and assessed at the 5% significance level. Sensitivity analyses will be performed to confirm that clustering makes negligible difference to results and no difference to conclusions or interpretations, using Donner's Method [27]. Hypotheses II and III will be assessed by comparing the primary outcome between mutation status subgroups of the DNA arm with each other and with the control arm. To strengthen the interpretation of these non-randomised comparisons, sensitivity analysis will be undertaken to vary the degree to which mutation status may predict outcome, detailed as part of the statistical analysis plan to be prepared prior to analysis.

### Design considerations

We considered a design in which saliva samples are taken in both trial arms. The potential advantage of this design is that it increases the similarity between trial arms for procedures of no pure experimental interest and allows the distribution of genotypes in each arm to be assessed in order to allow adjustment for any imbalance in the analysis. The potential disadvantage of such a design is that the taking of a saliva sample in those whose DNA is not being used to estimate their risk of CD may lead to a sense of having lost out on randomisation, and that taking a sample for analysis can thus be seen as an integral part of the DNA assessment and communication intervention as applied in practice. We judged this disadvantage to outweigh the benefits of such a design and thus rejected it for use in the trial.

## Trial details

Registration: ISRCTN21633644

Date trial started: April 2007

Expected end date: September 2010

Expected reporting date: February 2011

## Competing interests

The authors declare that they have no competing interests.

## Authors' contributions

TMM, DA, ALK, ATP, and SS are the Principal Investigators for the trial. ATP is the trial statistician and SCLW and GJH are the trial managers. CM and CL are responsible for DNA analysis and calculation of risk figures, AF and JS provided methods for participant recruitment, and SW developed the counselling protocol. All authors drafted the manuscript and read and approved the final version. TMM is the paper guarantor.

## Pre-publication history

The pre-publication history for this paper can be accessed here:

http://www.biomedcentral.com/1471-2458/11/44/prepub
